# An SSL-PUF Based Access Authentication and Key Distribution Scheme for the Space–Air–Ground Integrated Network

**DOI:** 10.3390/e25050760

**Published:** 2023-05-06

**Authors:** Liwei Xu, Han Wu, Jianguo Xie, Qiong Yuan, Ying Sun, Guozhen Shi, Shoushan Luo

**Affiliations:** 1 School of Cyberspace Security, Beijing University of Posts and Telecommunications, Beijing 100876, China; xuliwei@bupt.edu.cn; 2 Beijing Electronic Science and Technology Institute, Beijing 100070, China; 3 Wuhan Maritime Communication Research Institute, Wuhan 430205, China

**Keywords:** PUF, SAGIN, semiconductor superlattice, authentication, key distribution

## Abstract

The Space–Air–Ground Integrated Network (SAGIN) expands cyberspace greatly. Dynamic network architecture, complex communication links, limited resources, and diverse environments make SAGIN’s authentication and key distribution much more difficult. Public key cryptography is a better choice for terminals to access SAGIN dynamically, but it is time-consuming. The semiconductor superlattice (SSL) is a strong Physical Unclonable Function (PUF) to be the hardware root of security, and the matched SSL pairs can achieve full entropy key distribution through an insecure public channel. Thus, an access authentication and key distribution scheme is proposed. The inherent security of SSL makes the authentication and key distribution spontaneously achieved without a key management burden and solves the assumption that excellent performance is based on pre-shared symmetric keys. The proposed scheme achieves the intended authentication, confidentiality, integrity, and forward security, which can defend against masquerade attacks, replay attacks, and man-in-the-middle attacks. The formal security analysis substantiates the security goal. The performance evaluation results confirm that the proposed protocols have an obvious advantage over the elliptic curve or bilinear pairings-based protocols. Compared with the protocols based on the pre-distributed symmetric key, our scheme shows unconditional security and dynamic key management with the same level performance.

## 1. Introduction

The Space–Air–Ground Integrated Network (SAGIN) is a heterogeneous network architecture consisting of satellite, aerial, and territorial segments [1]. Integrating different networks expands cyberspace from the traditional internet to the land, sea, sky, and outer space, making SAGIN vital for strategic importance [2]. Space information is a crucial point of society and cyberspace. Thus, the security of space information has to be strengthened [3]. As the first line of network defense, access authentication and key distribution schemes are crucial technology to prevent illegal terminals for the security of SAGINs [4].

The high mobility, low latency, and limited resource in SAGIN have put forward higher requirements for security issues, such as identity authentication and data security [5]. Besides, access at any time means high-quality network services, such as dynamic access and a smooth handover authentication mechanism caused by the movement of satellites.

A lightweight authentication protocol has been proposed based on different techniques. Public key cryptography is suitable for dynamic terminals to access SAGIN. Schemes based on elliptic curve cryptography and symmetric keys to provide anonymity and authentication were proposed [6,7,8,9,10]. However, the operation over the elliptic curve is time-consuming. Some schemes based on the symmetric key, secret sharing, or group key are lightweight and need less communication overhead [11,12,13]. Nevertheless, the pre-shared symmetric key is always the key point in modern cryptography, and key distribution and management are the foundation of cryptography. The assumption that many different terminals pre-share symmetric keys with satellites or ground servers is unrealistic.

In order to reduce the exchange procedure, a solution with bilinear pairings was proposed, but it is resource-consuming for the terminal and satellites [14,15,16]. As the privacy protection requirements, a lightweight authentication protocol was proposed based on 3GPP AKA for the fast grouping of the satellites [17]. An improved authentication method based on tokens was proposed, which can provide the anonymity of the terminal but still did not reduce the burden on the management center [18]. The blockchain-based on hash is an alternative scheme to reduce the complexity of negotiation among different terminals and regions [19,20,21,22]. However, the construction of the public or private blockchain makes SAGIN much more complicated.

Unlike public key cryptography, which is computational security, Quantum Key Distribution (QKD) is based on the uncertainty principle of quantum physics [23]. QKD offers the ultimate solution: restoring security and confidentiality by resorting to unbreakable principles of nature [24]. Discrete-Variable QKD (DVQKD) and Continuous-Variable QKD (CVQKD) are representative QKD protocols. The measurement-device-independent protocol has closed the critical side channels in the physical implementations, paving the way for secure DVQKD with realistic devices [25]. CVQKD is based on coherent states or Gaussian modulation focuses on practical implementations [26,27,28]. However, the complex equipment in QKD is not suitable for diverse terminals in SAGIN.

Regarding physical security, several authentication protocols based on Physical Unclonable Function (PUF) have been proposed [29,30,31,32,33]. The inherent unclonable function makes PUF a physical one-way function suitable as physical security primitives [34,35]. The challenge–response pairs (CRPs) are exchanged in the registration procedure, and the authentication and key exchange protocols are formalized based on the CRPs. The attacker cannot simulate the correct CRPs without the registered PUF. However, in SAGIN, there are numerous terminals versus a certain number of satellites. It is not realistic to store all the CRPs of all the terminals in one satellite, let alone the handover situation of the satellites.

Semiconductor superlattice (SSL) is a strong PUF that consists of two different materials [36]. Generally speaking, SSL is employed in authentication or random number generation.

Besides, the chaos synchronization between unclonable matched superlattice pairs in the same wafer was discovered. A long-haul public-channel secure key distribution was experimentally demonstrated based on matched SSL pairs [37,38]. Matched SSL pairs can also be employed to distribute symmetric keys even if in different locations, such as Beijing and Changsha. Furthermore, the full entropy keys can be distributed through an insecure public channel, greatly reducing the complexity of key distribution and management.

In conclusion, a public key over the elliptic curve achieves dynamic access for SAGIN. However, it is more time-consuming than a lightweight authentication scheme based on a pre-shared symmetric key. However, how to achieve secure and convenient symmetric key distribution and management is a relatively difficult problem, especially in high mobility SAGIN. SSL PUF is suitable to protect from a physical attack. Furthermore, the matched SSL pairs are a novel scheme for unconditional key distribution dynamically through a public channel. The SSL-based access authentication and key distribution scheme is proposed for SAGIN. The major contributions of this paper are described below:

1. A system model for access and key distribution based on SSL PUF is proposed. In the system model, various terminals are equipped with regular SSL chips. Meanwhile, satellites and the ground server are equipped with matched SSL pairs. The inherent security of SSL makes the authentication and key distribution spontaneously achieved without a key management burden and solves the assumption that excellent performance is based on pre-shared symmetric keys.
2. Access authentication and handover authentication schemes are proposed, which achieve mutual access authentication and unconditionally secure key distribution. The protocol maintains a lower cost and communication overhead by employing basic *hash/hmac* operations and symmetric encryption.
3. The proposed scheme achieves intended authentication, confidentiality, integrity, and forward security, which can defend against masquerade attacks, replay attacks, and man-in-the-middle attacks. The informal security analysis substantiates the security goal.
4. The performance evaluation results confirm that the proposed protocols have an obvious advantage over the elliptic curve or bilinear pairings-based protocols. Compared with the protocols based on a pre-distributed symmetric key, our scheme shows unconditional security and dynamic key management versus a somewhat weaker performance.

In Section 2, background on the SSL is introduced. Section 3 describes the system model and security goals. The proposed scheme is illustrated in Section 4, and the security analysis is given in Section 5. Performance evaluation is in Section 6. The conclusion is the final part of Section 7.

## 2. Background on the Semiconductor Superlattice

### 2.1. Semiconductor Superlattice (SSL) PUF

**Figure 1:**
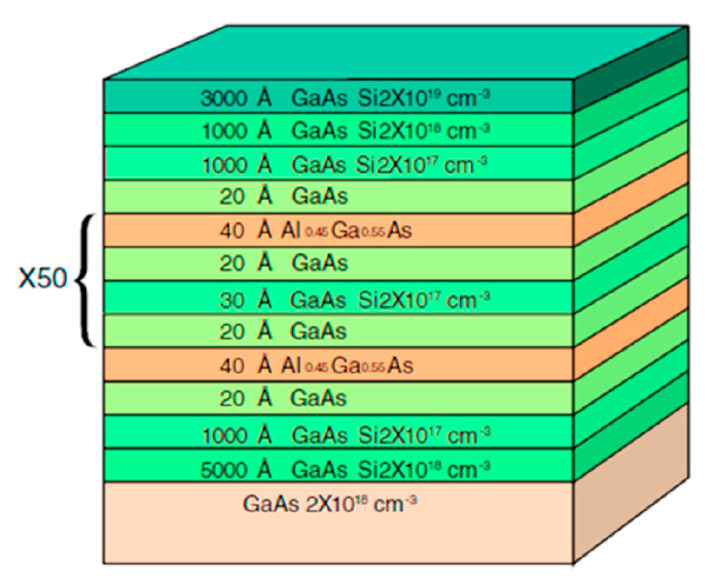
Schematic of SSL $GaAs/Al_{0.45}Ga_{0.55}As$ [36].

The semiconductor superlattice (SSL) is an artificial, periodic, multilayer semiconductor material, which is composed of nanoscale materials $GaAs/Al_{0.45}Ga_{0.55}As$. The schematic of SSL is shown as Figure 1.

**Figure 2:**
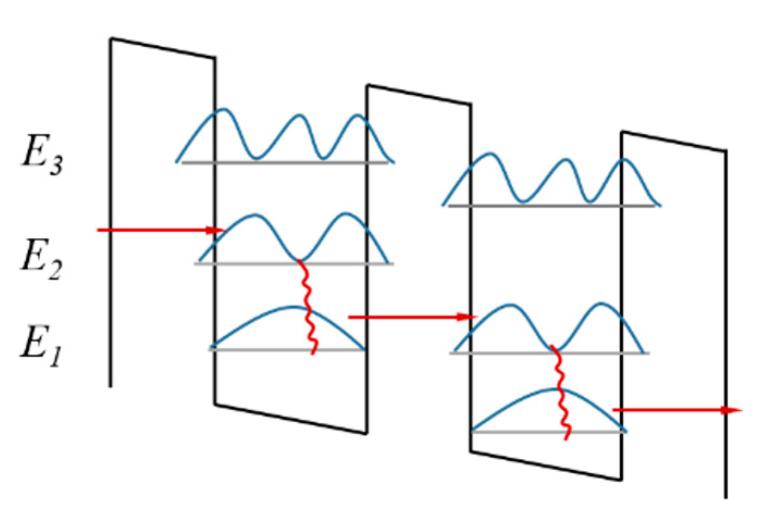
Schematic of quantum resonant tunnelling.

Under a certain bias voltage range, SSL shows a nonlinear chaotic status with multiple degrees of freedom due to the quantum resonance tunneling effect. Quantum resonance tunneling satisfies both energy conservation and wave vector conservation. The red lines in Figure 2 shows the electronics moving across different energy levels while quantum resonance tunneling occurs. The behavior of electronics travelling through the quantum wells are unpredictable in quantum resonance tunneling. The energy levels are extremely sensitive to the nanoscale layers $GaAs/Al_{0.45}Ga_{0.55}As$, which contains millions of atoms. It is impossible to manipulate SSL atom to atom, which means that SSL is physically unclonable and unpredictable. Simulating the structure of SSL mathematically is also impractical, even with a modest quantum computer [39,40].

The response signal is generated when SSL is inspired by a challenge signal, which makes SSL acts as a physical one-way function. SSL is a new chaotic material that works as a strong PUF [37], and a true random number generator (TRNG) was proposed based on SSL-PUF [36,38].

Strong PUF has the property that it is prohibitively hard to clone; a complete enumeration of all its CRPs is intractable [39]. Furthermore, an interesting characteristic of SSL is matched SSL pairs from the same wafer, which makes SSL suitable for full entropy key distribution techniques [37,41,42].

According to the definition in [34,43], PUF seems to be a one-way physical function that replies with a response corresponding to the challenge. Equation (1) shows the physical function of SSL-PUF; ${\mathrm{PUF}}_{SSL}$ dedicates a piece of SSL chip, c is the challenge, and r is the response. The detail mapping relation relies on the intrinsic structure introduced by the physical growth procedure, which is uncontrollable.

(1) $$R={\mathrm{PUF}}_{ssl}\left(C\right).$$

SSL also exhibits the unclonable and unpredictable properties of PUF, which are the security root of the PUF [43]. The matched pairs property gives SSL a higher chance in cryptography applications.

Unclonable: For a given SSL chip, ssl, and challenge, c, the corresponding $r={\mathrm{PUF}}_{ssl}\left(c\right)$. For the same challenge, c, the probability of finding another SSL chip, $ssl^{\prime }$, with the same response, r, is negligible, just as Equation (2) shows. Prob denotes probability, and $\varepsilon \leq 10^{-6}$. The ssl and $ssl^{\prime }$ are from different wafers, so they are unmatched.

(2) $$\mathrm{Prob}\left[{\mathrm{PUF}}_{ssl^{\prime }}\left(c\right)={\mathrm{PUF}}_{ssl}\left(c\right)\right]\leq \varepsilon .$$

**Unpredictable**: For any given SSL chip, ssl, the probability of predicting the response of any randomly selected challenge, $c^{\prime }$, is negligible, just as Equation (3) shows.

(3) $$\mathrm{Prob}\left[\mathrm{find}\;r\;\mathrm{and}\;r={\;\mathrm{PUF}}_{ssl}\left(c^{\prime }\right)\right]\leq \varepsilon .$$

**Matched pairs**: For SSL chips, $ssl_{1}$ and $ssl_{2}$, in the same wafer and very close to each other, they are called matched pairs when Equation (4) holds.

(4) $$\mathrm{Prob}\left[\mathrm{HD}\left({\mathrm{PUF}}_{ssl_{1}}\left(c\right),{\mathrm{PUF}}_{ssl_{2}}\left(c\right)\right)\leq 0.05l\;\right]\geq 1-\varepsilon .$$

For any challenge, c, the responses of matched pairs, $ssl_{1}$ and $ssl_{2}$, are nearly the same, and only about 5% of the responses are different. HD denotes the Hamming Distance (HD), and l denotes the bit length of response. Matched pairs are inspected and tested strictly, and the little difference can be erased by Information Reconciliation technology in cryptography [34]. Long-haul key distribution based on matched pairs can be performed, and the key distribution arguments can be transported in the public channel even if the matched pairs are in a different city [42,44].

### 2.2. SSL Authentication and Key Distribution

The challenge mainly employs the authentication and key distribution scheme based on PUF and response pairs (CRPs) exchanged between the terminal and the authentication server [33,45,46,47,48]. Since SSL’s have unclonable and unpredictable properties, as the other PUFs, the CRPs for a certain SSL chip are fixed and unclonable to fake. The Ground Server (GS) pre-stores the CRPs of the SSL chip, and the Terminal with the SSL chip will send a challenge as an authentication message. The terminal authenticates successfully to the server with the same and nearly fixed response. “Nearly fixed” here is caused by the analog signal of the PUF response inevitably has a tiny difference for the same challenge [49]. However, the difference can be wiped out by Fuzzy Extractors, which usually contain Information Reconciliation (IR) and Privacy Amplification (PA) [49,50]. The IR corrects the analog deviation, and PA extracts sufficient information as a key used in cryptography.

**Figure 3:**
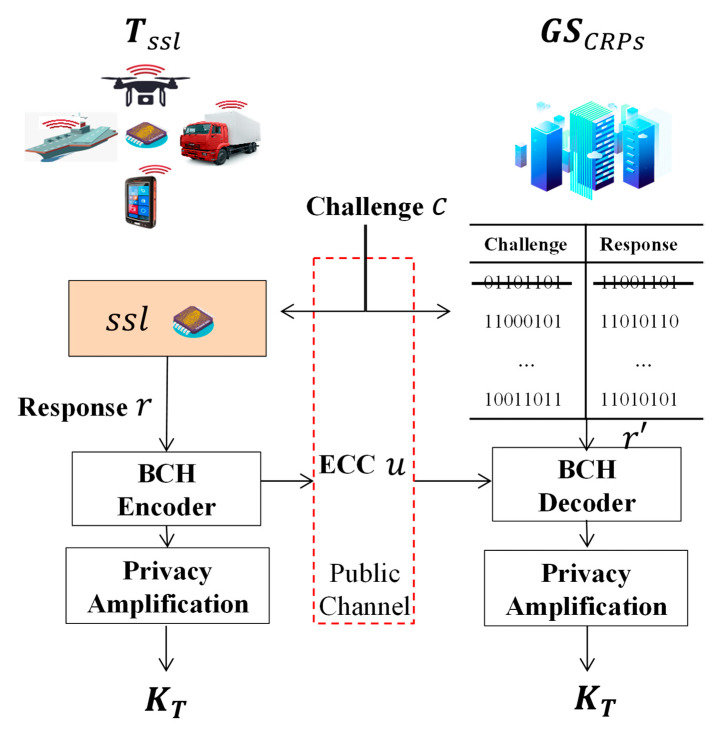
Overview of the SSL key distribution scheme.

Figure 3 shows the key distribution procedure. The terminal selects a challenge, c, and the SSL outputs the response, r. The BCH (Bose, Ray Chaudhuri, and Hocquenghem) code is used as an IR procedure, which is efficient for error correcting code [49,51]. The Error Correcting Code (ECC), u, is sent to GS instead of the response, r, which has redundant information of *r*. The challenge, c, is sent to GS through the public channel too. The pre-stored $r^{\prime }$ is selected from the database of GS and corrected by ECC, u. Finally, the key between the Terminal and GS is extracted by Privacy Amplification [49,50,52]. The related response, $r^{\prime }$, can be used only once to avoid a replay attack.

### 2.3. Matched SSL Pairs for Key Distribution

**Figure 4:**
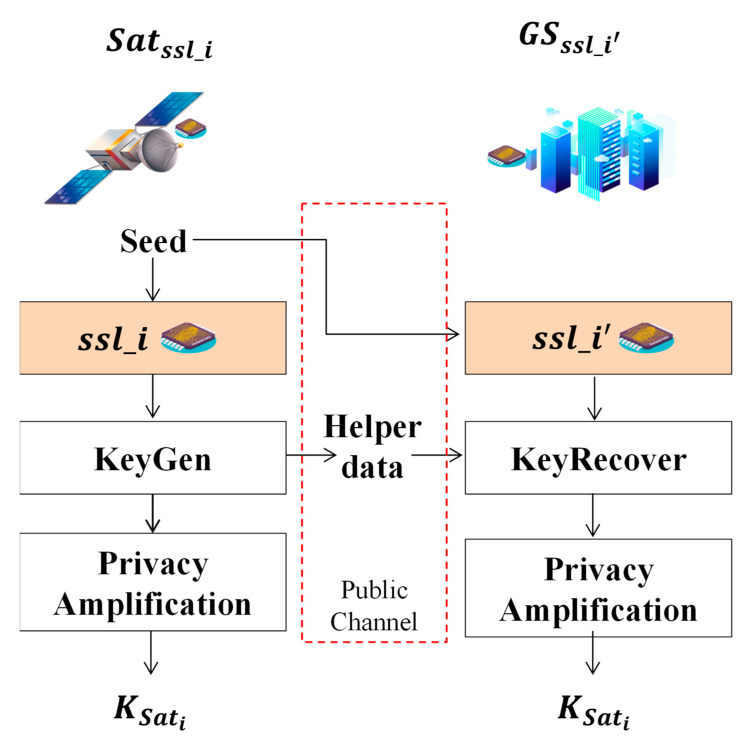
Overview of the key distribution scheme of matched SSL pairs.

Compared to the regular SSL PUF, the key distribution scheme is simple and clear for matched SSL pairs, as shown in Figure 4. A SSL PUF chip, ${\mathrm{ssl}}_{i}^{\prime }$, matched to ${\mathrm{ssl}}_{i}$ is installed in GS that has a similar response with the satellite, ${\mathrm{Sat}}_{{\mathrm{ssl}}_{i}}$, inspired by the same seed [49,52]. The KeyGen procedure generates helper data publicly sent together with the seed to associate the Key Recover procedure. The KeyGen and Key Recover procedure correspond to the BCH Encoder and Decoder algorithm. The final result, $K_{{\mathrm{Sat}}_{I}}$, can be a key buffer pool for many symmetric keys. However, in this paper, we use $K_{{\mathrm{Sat}}_{i}}$ in short. All the information used for key distribution can be delivered publicly, which is a fascinating advantage compared to the other key distribution scheme.

## 3. System Model and Security Requirements

### 3.1. System Model

Derived from the Internet of Things (IoT), SAGIN is developed based on the Vehicular Ad-hoc Network (VANET) and Maritime Communication Network (MCN) [1,14,53,54]. Various terminals, such as mobile phones, traffic terminals, vessels with sensors, and Unmanned Aerial Vehicles (UAVs), are working in various practical scenarios where they cannot always connect to the network services. The satellites broaden the communication of terminals to the global coverage. The terminals join the SAGIN through access authentication and have to deal with the handover authentication among the satellites.

**Figure 5:**
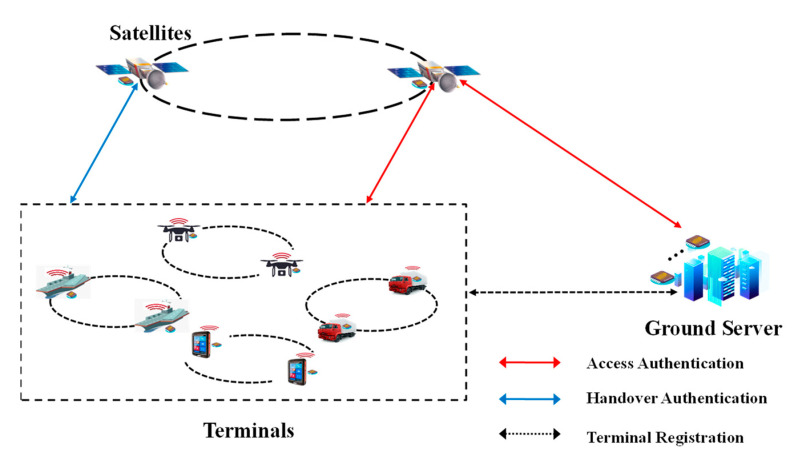
System model for authentication.

Figure 5 shows this paper’s system model, consisting of a terminal, satellite, and ground server (GS). Every terminal is equipped with a SSL PUF as a unique physical identification, and the CRPs of the terminal are generated in the registration procedure. By the CRPs pre-stored in the GS, terminals accompany the access authentications through the satellites. Since the satellite network is changing in space, the terminal has to deal with the handover authentication with satellites. Match SSL PUF pairs are installed in the satellite and GS, which accompany the access authentication and build the secure communication channel between them.

### 3.2. Security Assumptions and Goals

In this paper, GS is assumed to be completely trustful, which means the CRPs of all the terminals are safely stored and used. The registration of the terminal is carried out in a secret channel. Moreover, the satellites are safe in the air and cannot be stolen. According to the Dolev–Yao Model, it is assumed that the adversary has the ability [55]. The interaction of the protocol in the air can be inspected, modified, or interrupted by the adversary. Thus, the proposed scheme should fulfill the following security goals:

1. **Mutual Access Authentication**: The terminals, satellites, and GS can achieve mutual access authentication with each other;
2. **Handover Authentication**: When the terminal inspects that the satellite communicates with the terminal and is going to move outside the service range, a handover authentication should react smoothly;
3. **Key Distribution**: The scheme proposed should distribute a secret session key for each participant in the authentication procedure;
4. **Against regular security attacks and forward security**: The scheme proposed should defend against masquerade attacks, replay attacks, man-in-the-middle attacks, and have forward security if the terminal with the SSL PUF is ever stolen.

Above all, the proposed scheme should have authentication, confidentiality, integrity, and forward security.

## 4. The Proposed Scheme

The proposed scheme consists of three participants: terminal T, satellites Sat, and ground server GS. The scheme is divided into three phases: terminal registration, authentication, and handover. Table 1 shows the notations used in this paper.$\;GS_{CRPs}$ and $GS_{ssl\_i^{\prime }}$ are the same GS, and are distinguished for easy understanding.

### 4.1. Terminal Registration Phase

The most important work in the terminal’s registration phase is generating and storing the terminal’s CRPs in GS. It is assumed that the GS is in a safe environment, and the registration procedure is executed in a secret channel.

Let N be the number of CRPs according to the application.

1. $GS_{CRPs}$ select a random number as the starting point of the challenge, $c_{start}$, and save it in the database, then set the challenge $c=c_{start}$, and send c to terminal $T_{ssl}$.
2. $T_{ssl}$ saves $c_{start}=c$ as a starting point of the challenge. Inspire the SSL PUF chip by c, and get the response, r. Then send r to $GS_{CRPs}$.
3. $GS_{CRPs}$ save (Hash(TID||c),r) to the database. Then calculate $c=Hash\left(c\right)$, and send new challenge, c, to $T_{ssl}$.
4. $T_{ssl}$ get the response, r, of the challenge, c, and return r to $GS_{CRPs}$.
5. Execute step (3)~step (4) iteratively to get all N groups CRPs.

Finally, $T_{ssl}$ saves the initial value of challenge $c_{start}$, and $GS_{CRPs}$ saves $c_{start}$ and N groups CRPs of $T_{ssl}$. Hash(TID||c) is transmitted and stored instead of c because the Hash(TID||c) will keep the forward security of the scheme. Even if $T_{ssl}$ was stolen, the attacker would not get the correct response, r, without the correct c.

### 4.2. Authentication Phase

The access authentication phase consists of satellite access authentication and terminal access authentication. The satellite access authentication realizes the secure key distribution based on matched SSL pairs, and the key buffer can be established in advance, reducing the communication overhead and improving efficiency. Terminal access authentication is implemented based on the common SSL. The procedures of the two phases are described below.

#### 4.2.1. Satellite Access Authentication

**Figure 6:**
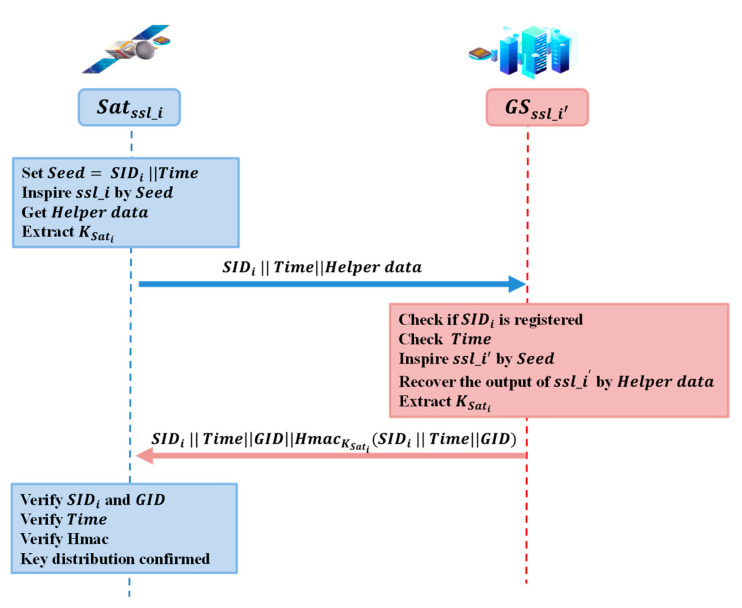
Satellite access authentication protocol.

Satellite $Sat_{ssl\_i}$ and ground server $GS_{ssl\_i^{\prime }}$ are equipped with matched SSL pairs, ssl_i and $ssl\_i^{\prime }$. The access authentication procedure is shown in Figure 6.

1. $Sat_{ssl\_i}$ set $Seed=SID_{i}\;||\;Time$, and inputs it to the Sequence Synchronization module, which produces challenge signals continuously to ssl_i. The BCH mode processes the output sequence, and the Helper data is generated. Finally, the privacy amplification module extracts the symmetric key, $K_{Sat_{i}}$, from the output sequence. $Sat_{ssl\_i}$ send $SID_{i}\;\left|\left|\;Time\right|\right|Helper\;data$ to $GS_{ssl\_i^{\prime }}$ publicly.
2. $GS_{ssl\_i^{\prime }}$ checks the $SID_{i}$ to verify the access authentication of the satellite and checks the Time to avoid a replay attack. Then, $GS_{ssl\_i^{\prime }}$ gets nearly the same output sequence through $ssl\_i^{\prime }$, recovered by the Helper data. The secret key $K_{Sat_{i}}$ is distributed after the privacy amplification module. $GS_{ssl\_i^{\prime }}$ sends message $SID_{i}\;\left|\left|\;Time\right|\left|GID\right|\right|Hmac_{K_{Sat_{i}}}\left(SID_{i}\;\left|\left|\;Time\right|\right|GID\right)$.
3. $Sat_{ssl\_i}$ gets the message and checks $Hmac_{K_{Sat_{i}}}\left(SID_{i}\;\left|\left|\;Time\right|\right|GID\right)$ by the secret key, $K_{Sat_{i}}$, to confirm the key distribution protocol.

The secret key, $K_{Sat_{i}}$, can also be a large key buffer that can be prepared as soon as the system is started. Thus, the satellite access authentication procedure will not cost much regarding calculation and communication consumption.

#### 4.2.2. Terminal Access Authentication

**Figure 7:**
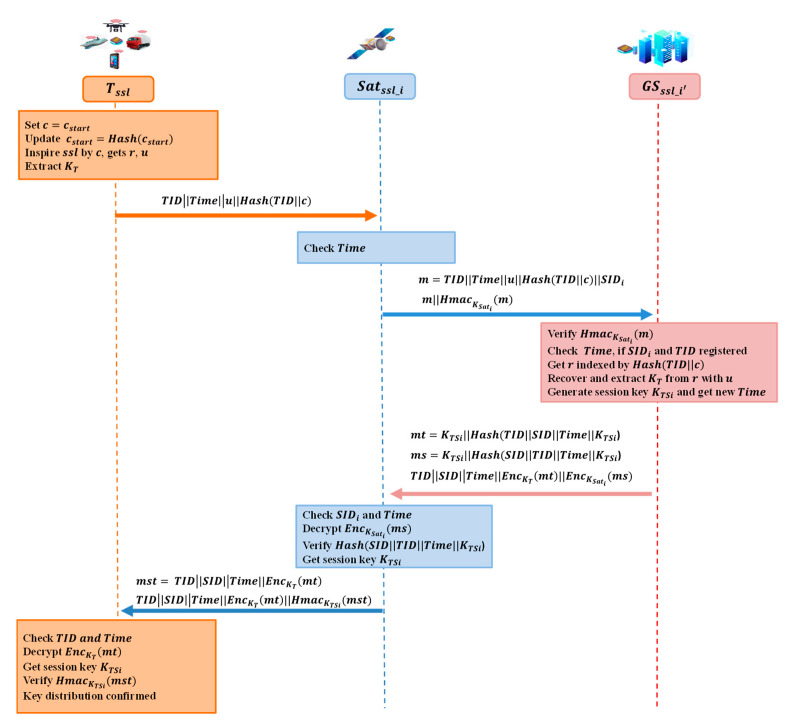
Terminal access authentication protocol.

In the terminal access authentication protocol, an SSL PUF chip is equipped in terminal $T_{ssl}$, and its CRPs are stored in the ground server $GS_{CRPs}$ in the registration procedure.$\;Sat_{ssl\_i}$ serves as a transmitter in the protocol. $GS_{CRPs}$ and $GS_{ssl\_i^{\prime }}$ are the same ones, called GS in short. The $Sat_{ssl\_i}$ and $GS_{ssl\_i^{\prime }}$ have established a secret channel before the terminal access authentication. Figure 7 shows the detailed processes.

1. Terminal $T_{ssl}$ finds the starting point of challenge $c_{start}$, sets $c=c_{start}$, and updates $c_{start}=Hash\left(c_{start}\right)$. Then, it inspires ssl by challenge, c, and gets the response, r, error correct code, u. Then, $K_{T}$ is extracted from the response, r. The terminal $T_{ssl}$ sends a message $TID\left|\left|Time\right|\right|u||Hash(TID||c)$ to the satellite, $Sat_{ssl\_i}$.
2. The satellite $Sat_{ssl\_i}$ checks the Time first to avoid a replay attack. Let message $m=TID\left|\left|Time\right|\right|u||Hash(TID\left|\left|c\right)\right||SID_{i}$. Satellite $Sat_{ssl\_i}$ sends $m||Hmac_{K_{Sat_{i}}}\left(m\right)$ to the GS.
3. The ground server, GS, verifies $Hmac_{K_{Sat_{i}}}\left(m\right)$ with $K_{Sat_{i}}$ first, and then checks the Time to avoid a replay attack. $SID_{i}$ and TID are checked if they were registered. Then, the pre-stored (Hash(TID||c), r) was indexed by Hash(TID||c), and $K_{T}$ between $T_{ssl}$ and $GS_{CRPs}$ is extracted according to Figure 3 with ECC, u. Mark the index Hash(TID||c) to avoid a replay attack.
4. The ground server, GS, generates a random number as the session key $K_{TSi}$ and gets the Time. Let message $mt=K_{TSi}||Hash(TID\left|\left|SID\right|\left|Time\right|\right|K_{TSi}$). Let message $ms=K_{TSi}||Hash(SID\left|\left|TID\right|\left|Time\right|\right|K_{TSi}$). GS sends $TID\left|\left|SID\right|\right|Time\left|\left|Enc_{K_{T}}\left(mt\right)\right|\right|Enc_{K_{Sat_{i}}}\left(ms\right)$ to Satellite $Sat_{ssl\_i}$.
5. Satellite $Sat_{ssl\_i}$ checks SID and Time first. Then, $Enc_{K_{Sat_{i}}}\left(ms\right)$ is decrypted by $Sat_{ssl\_i}$, the integrity of $Hash(SID\left|\left|TID\right|\left|Time\right|\right|K_{TSi}$) is verified. $Sat_{ssl\_i}$ gets the session key $K_{TSi}$. Let $mst=TID\left|\left|SID\right|\right|Time||Enc_{K_{T}}\left(mt\right)$. $Sat_{ssl\_i}$ sends $TID\left|\left|SID\right|\right|Time\left|\left|Enc_{K_{T}}\left(mt\right)\right|\right|Hmac_{K_{TSi}}\left(mst\right)$ to terminal $T_{ssl}$.
6. Terminal $T_{ssl}$ checks TID and Time first. Then, $T_{ssl}$ decrypts $Enc_{K_{T}}\left(mt\right)$ with $K_{T}$ and gets the session key $K_{TSi}$. Then, $Hmac_{K_{TSi}}\left(mst\right)$ is verified with $K_{TSi}$. Terminal access authentication and key distributed are verified.

### 4.3. Handover Authentication Phase

**Figure 8:**
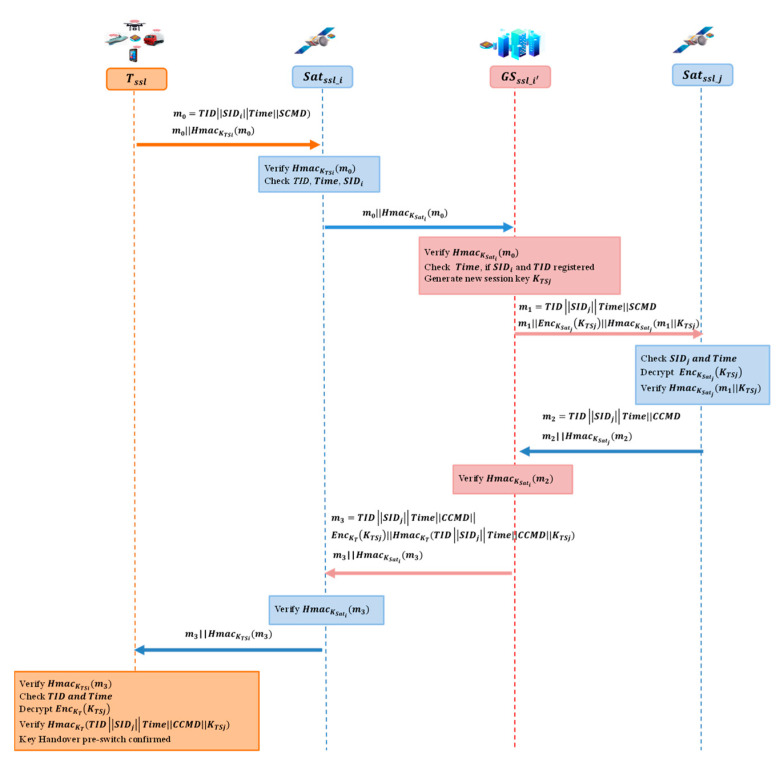
Handover authentication protocol.

Since the satellites are switching around in the air space, the handover authentication is considered to provide continuous network service to terminals on the ground [8,12]. A pre-switch handover authentication protocol is proposed based on the SSL, as Figure 8 shows. Terminal $T_{\mathrm{ssl}}$ can accomplish the pre-switch procedure before the handover switch so that the communication service is switched smoothly.

1. When the terminal, $T_{ssl}$, inspects that the satellite, $Sat_{ssl\_i}$, is going away from the service coverage, it sends a pre-switch request to $Sat_{ssl\_i}$. Let $m_{0}=TID\left|\left|SID_{i}\right|\right|Time||SCMD$. Sends message $m_{0}||Hmac_{K_{TSi}}\left(m_{0}\right)$.
2. $Sat_{ssl\_i}$ verifies $Hmac_{K_{TSi}}\left(m_{0}\right)$ with $K_{TSi}$, then checks TID, Time, and $SID_{i}$. Then, $Sat_{ssl\_i}$ sends $m_{0}||Hmac_{K_{Sat_{i}}}\left(m_{0}\right)$.
3. The ground server, GS, verifies $Hmac_{K_{Sat_{i}}}\left(m_{0}\right)$ with $K_{Sat_{i}}$. Then checks TID, Time, and $SID_{i}$. Next, GS calculates the next satellite, $Sat_{ssl\_j}$, to server the terminal $T_{ssl}$. GS generates a new session key, $K_{TSj}$, randomly. Let $m_{1}=TID\left|\left|SID_{j}\right|\right|Time||SCMD.$ Then, it sends $m_{1}\left|\left|Enc_{K_{Sat_{j}}}\left(K_{TSj}\right)\right|\right|Hmac_{K_{Sat_{j}}}(m_{1}||K_{TSj})$ to $Sat_{ssl\_j}$.
4. $Sat_{ssl\_j}$ checks $SID_{j}$ and Time, and decrypts $Enc_{K_{Sat_{j}}}\left(K_{TSj}\right)$ with $K_{Sat_{j}}$. Then, it verifies $Hmac_{K_{Sat_{j}}}(m_{1}||K_{TSj})$. Let $m_{2}=TID\left|\left|SID_{j}\right|\right|Time||CCMD$. A confirmation message $m_{2}$||$Hmac_{K_{Sat_{j}}}\left(m_{2}\right)$ is sent back to GS.
5. GS verifies $Hmac_{K_{Sat_{j}}}\left(m_{2}\right)$ and sends $m_{3}$||$Hmac_{K_{Sat_{i}}}\left(m_{3}\right)$ to $Sat_{ssl\_i}$ where $m_{3}=TID\left|\left|SID_{j}\right|\right|Time\left|\left|CCMD\right|\right|Enc_{K_{T}}\left(K_{TSj}\right)||Hmac_{K_{T}}\left(TID\left|\left|SID_{j}\right|\right|Time\left|\left|CCMD\right|\right|K_{TSj}\right)$.
6. $Sat_{ssl\_i}$ verifies $Hmac_{K_{Sat_{i}}}\left(m_{3}\right)$ and sends $m_{3}$||$Hmac_{K_{TSi}}\left(m_{3}\right)$ to terminal $T_{ssl}$.
7. Terminal, $T_{ssl}$, verifies $Hmac_{K_{TSi}}\left(m_{3}\right)$ with *K_TSi_*, checks TID and Time, decrypts $Enc_{K_{T}}\left(K_{TSj}\right)$ with $K_{T}$, and verifies. $Hmac_{K_{T}}\left(TID\left|\left|SID_{j}\right|\right|Time\left|\left|CCMD\right|\right|K_{TSj}\right)$. The pre-switch protocol is finished.

## 5. Security Analysis

### 5.1. Informal Security Analysis

#### 5.1.1. Mutual Authentication

The mutual authentication between the satellite, $Sat_{ssl\_i}$, and ground server, $GS_{ssl\_i^{\prime }}$, relays on the matched SSL pairs ssl_i and $ssl\_i^{\prime }$. According to Equation (4), only the matched SSL pairs can achieve the same session key with the same Seed and publicly transfer Helper data.

In terminal access authentication, the terminal, $T_{ssl}$, and ground server, $GS_{ssl\_i^{\prime }}$, authenticate each other based on the pre-stored CRPs in $GS_{ssl\_i^{\prime }}$. Only the corresponding terminal with the correct SSL chips can authenticate with $GS_{ssl\_i^{\prime }}$. Concerning terminal, $T_{ssl}$, and satellite, $Sat_{ssl\_i}$, the same symmetric secret key, $K_{TSi}$, is the key point of authentication.

$Hmac_{K_{TSi}}\left(mst\right)$ can be verified successfully by the legitimate $Sat_{ssl\_i}$ with the same $K_{TSi}$ that the $GS_{ssl\_i^{\prime }}$ distributes.

The new satellite, $Sat_{ssl\_j}$, authenticates with GS in the handover authentication scenario based on the matched SSL pairs. Terminal, $T_{ssl}$, authenticates with GS based on the symmetric key, $K_{T}$, between them.

#### 5.1.2. Key Distribution

Similar to the mutual authentication scheme, the key distribution scheme employs the matched SSL pairs and pre-stored CRPs to accomplish the key distribution function. Only the legitimate terminal or satellite with the corresponding SSL PUF chips will achieve the symmetric secret key. Attackers without the SSL chips cannot recover the secret key successfully.

#### 5.1.3. Against Masquerade Attack

Unclonable SSL PUF makes the masquerade attack impossible. The cost and resource to clone or fake certain SSL chip is enormous, which make it is impossible [35].

#### 5.1.4. Against Replay Attack

The proposed scheme used Time to avoid replay attacks in the authentication and key distribution protocols. Furthermore, Time is added to the integrity by Hash or Hmac. In addition, the GS marks the used index Hash(TID||c) in case a replay authentication messes up the system. The replay attack can be detected by the authentication code or timestamp validation.

#### 5.1.5. Against Man-in-the-Middle Attack

A man-in-the-middle attacker without SSL chips cannot generate the correct response, r, nor recover the correct symmetric secret key, $K_{Sat_{i}}$, or $K_{T}$. The attacker cannot fake the Hmac or decrypt the session key $K_{TSi}$, so no one can play the middle man in the protocol.

#### 5.1.6. Forward Security

If terminal, $T_{ssl}$, was hijacked, the attacker gets the legal SSL chip. In this scenario, the correct response will be captured by the attacker too. In our scheme, the terminal sends Hash(TID||c) instead of the exact challenge, c, as the index of the CRPs in the GS. According to the one-way function property of Hash, the attacker cannot get the correct challenge, c, from Hash(TID||c). Thus, even if $T_{ssl}$ was stolen, the attacker cannot get the correct response, r, without the correct challenge, c. The attacker cannot recover the forward messages, which are encrypted by response, r.

#### 5.1.7. Quantum Computing Threat

Modern cryptographic systems need to be prepared to withstand the threats posed by the era of quantum computing. The SSL-PUF belongs to physical cryptography just as the quantum key distribution scheme does. The movement of the electronics in SSL-PUF is unpredictable, and the behaviors are extremely sensitive to the nanoscale layers $GaAs/Al_{0.45}Ga_{0.55}As$, which contains millions of atoms. Therefore, it is impossible to copy the exact SSL-PUF chip. Simulating the structure of SSL mathematically is also impractical, even with a modest quantum computer [39,40].

### 5.2. Formal Security Analysis

Since the satellite access authentication between the satellite and ground station relies on the matched SSL pairs, which can be seen as matched keys physically, the attackers could not fetch the correct session keys even if he has got the authentication information online.

The Handover authentication protocol is also the same principle. Therefore, the formal security analysis will focus on the terminal access authentication protocol.

The formal security analysis is employed by the Mao Boyd logic, which is improved on the Ban logic [56]. We use *T, Sat*, and *GS* to represent the terminal, satellite, and ground station.

Following the definitions and rules in [56], we generated the idealized protocol below:

1. *T* -> *Sat*: *TID*, *Time*.
2. *Sat* -> *GS*: *TID*, *SID*, *Time |*$\;Hmac_{K_{Sat_{i}}}\left(m\right)$.
3. *GS -> Sat*: *TID*, *SID*, ${\left\{K_{TSi}\;ℜ\;Time\right\}}_{K_{T}}\;{\left\{K_{TSi}\;ℜ\;Hmac_{K_{Sat_{i}}}\left(m\right)\right\}}_{K_{Sat_{i}}}$.
4. *Sat -> T*: *TID*, *SID*, ${\left\{TID,SID,K_{TSi}\;ℜ\;Time\right\}}_{K_{T}}$.

According to [56], unnecessary information on authentication is omitted. In message (1), *Time* is the challenge from *T* to *Sat*. $Hmac_{K_{Sat_{i}}}\left(m\right)$ together with *Time* in message (2) are the challenges from *Sat* to *GS*. The first $K_{TSi}$ in message (3) is the response to *Time*. The second $K_{TSi}$ is the response to $Hmac_{K_{Sat_{i}}}\left(m\right)$. The cipher of $K_{TSi}||Hash(TID\left|\left|SID\right|\left|Time\right|\right|K_{TSi}$) is sent in message (3), and the TID and SID are equivalently sent secretly. Note that the challenge and response are different from the concepts in PUF.

The assumptions of the protocol are (5)~(8):

(5) $$T|\equiv T\overset{K_{T}}{↔}GS.$$

(6) $$T|\equiv sup\left(GS\right).$$

(7) $$T|\equiv \#\left(Time\right).$$

(8) $$T\left|\equiv GS\right|\equiv {\left\{GS\right\}}^{C}⊲||K_{TSi}.$$

The goal of the formal analysis is to prove the statement “T believes $K_{\mathrm{TSi}}$ is a good secret between T and Sat”:

(9) $$T|\equiv T\overset{K_{TSi}}{↔}Sat.\;$$

**Figure 9:**
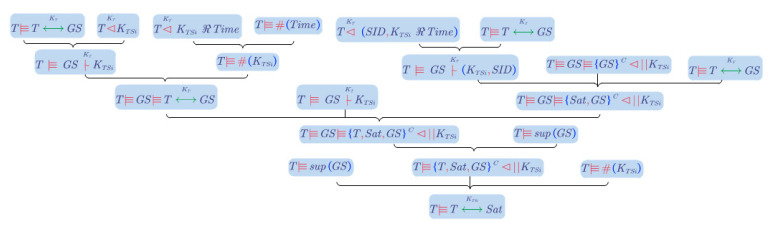
A tableau for demonstrating the procedure of proof.

The tableau for the procedure of proof is shown in Figure 9.

In message (4), we get statement (10):

(10) $$T\overset{K_{T}}{⊲}K_{TSi}.$$

According to **the authentication rules** with statements (5) and (10), statement (11) is deduced:

(11) $$T|\equiv GS\overset{K_{T}}{|\sim }K_{TSi}.$$

In message(4), terminal, *T*, sees the response to *Time*, as statement (12):

(12) $$T\overset{K_{T}}{⊲}K_{TSi}\;ℜ\;Time.\;$$

Applying **the fresh rules** to statements (12) and (7), we get statement (13):

(13) $$T|\equiv \#\left(K_{TSi}\right).$$

Statement (13) and (11) are deduced to statement (14) according to **the nonce-verification rules**.

(14) $$T\left|\equiv GS\right|\equiv T\overset{K_{T}}{↔}GS.$$

In another procedure, we get statement (15) from message (4)

(15) $$T\overset{K_{T}}{⊲}\left(SID,K_{TSi}\;ℜ\;Time\right).\;$$

Applying **the authentication rules** to statement (5) and (15), we get statement (16):

(16) $$T|\equiv GS\overset{K_{T}}{|\sim }\left(K_{TSi},SID\right).\;$$

Applying **the derived rules D2** to statements (5), (8), and (16), we get statement (17):

(17) $$T\left|\equiv GS\right|\equiv {\left\{Sat,GS\right\}}^{C}⊲||K_{TSi}.$$

Statements (14), (11), and (17) are deduced to statement according to **the derived rules D1**.

(18) $$T\left|\equiv GS\right|\equiv {\left\{T,Sat,GS\right\}}^{C}⊲||K_{TSi}.$$

Applying **the super-principal rules** to statements (18) and (62), we get statement (21):

(19) $$T\left|\equiv {\left\{T,Sat,GS\right\}}^{C}⊲\right||K_{TSi}.$$

At last, applying **the good-key rules** to statements (19), (6), and (8), we get statement (9):$T|\equiv T\overset{K_{TSi}}{↔}Sat$.

## 6. Simulation and Performance Evaluation

### 6.1. Simulation

**Figure 10:**
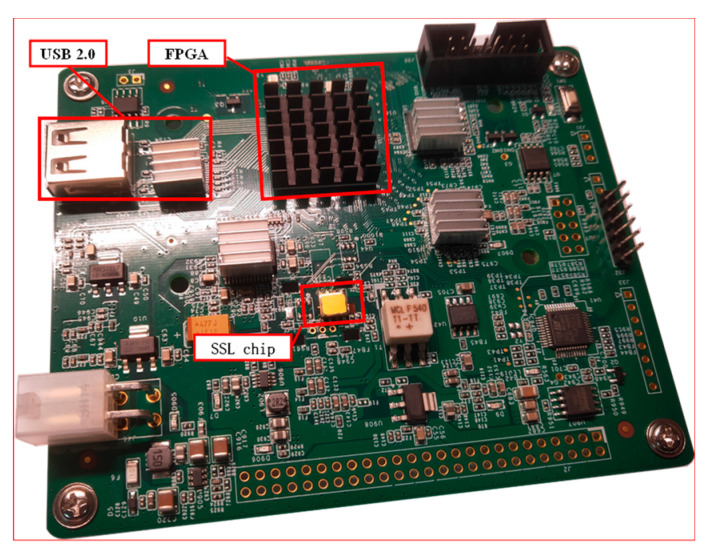
Simulation circuit board for terminal/satellite.

The simulation of regular PUF in the terminal or satellite is conducted on a standalone circuit board, as Figure 10 shows. The simulation circuit board transmits challenges and responses through a USB 2.0 port. The BCH encoder and decoder program run in a Field Programmable Gate Array (FPGA). A regular SSL chip is equipped in the circuit board to simulate the terminal and a matched SSL chip instead for satellite.

**Figure 11:**
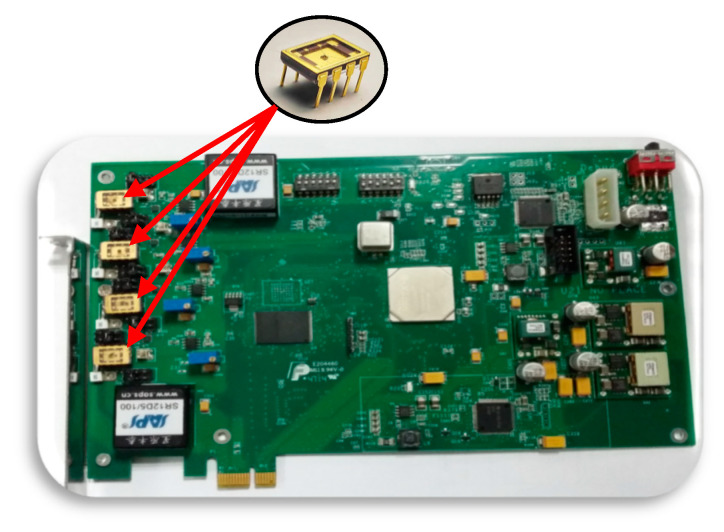
Simulation circuit board for the terminal/satellite.

The simulation for the GS is carried out on a circuit board, as shown in Figure 11. At the same time, four matched SSL pairs are equipped in the circuit board to simulate four satellites negotiating with GS. The simulation circuit board is designed by Suzhou Institute of Nano-tech and Nano-Bionics (SINANO), Chinese Academy of Sciences.

The performance of the proposed scheme is evaluated and compared in the computation overhead and communication overhead. Depending on the different emphasis, the performance of the access authentication is compared with the existing access authentication schemes, such as references [7,8,9,10,12]. The handover authentication scheme is compared with handover schemes in [8,11,12,14,18]. We choose SM4-128 bit [57] as the symmetric encryption algorithm, SM3 256 bit as the hash function, SM3-HMAC 256 bit [58] as Hmac, and set elliptic curve parameters as SM2 [59].

### 6.2. Computational Overhead

In order to evaluate the computational overhead, some typical operations are simulated and tested. Referring to [8], the terminal and satellite are simulated on Intel Core m3-6Y30 CPU@0.9 GHz, and the ground server is simulated on Intel Core i7-6600@2.6 GHz. The runtime costs are evaluated by library openssl-1.0.2e, and details are in Table 2. The $T_{puf}$, $T_{BCHE}$, $T_{BCHD}$, and$\;T_{PA}$ are special SSL PUFs, representing the cost of SSL response, BCH, and Privacy Amplification module. The computation cost of the compared schemes is calculated according to each protocol. Detail operation is abstracted from the protocol and accumulated based on the cost of each operation in Table 2.

**Figure 12:**
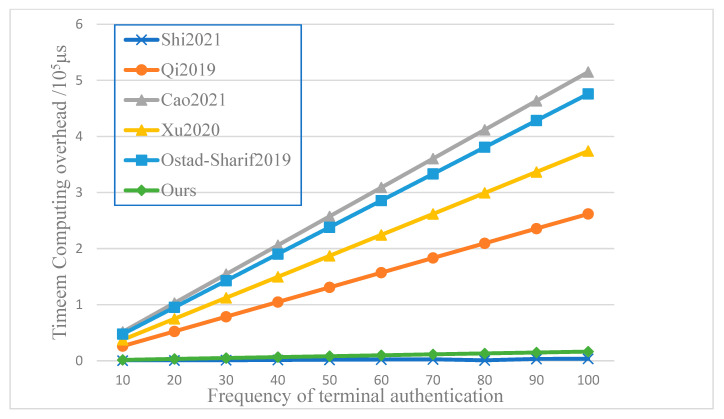
Comparison of terminal authentication cost [7,8,9,10,12].

The computation cost comparison of the terminal authentication scheme is in Table 3. The results show that our terminal authentication scheme has an obvious advantage compared with the scheme based on Elliptic Curve Cryptography [7,8,9,10] because the point multiplication over an elliptic curve is somewhat more time-consuming than hash and symmetric encryption, as shown in Figure 12. From the aspect of the total cost, our scheme is a little slower than the scheme based on a pre-distributed symmetric key [12], but still at the same level. However, the symmetric key distribution and management is cryptography’s most important and difficult point. Thus, our scheme, based on SSL PUF, has achieved the unconditional security key distribution with full entropy.

The computational cost comparison of handover authentication also shows a similar conclusion in Table 4. References [10,11,12] and our scheme are much faster than the scheme base on Elliptic Curve Cryptography [8] and bilinear pairings [14]. However, our scheme solved the key distribution problem properly and has a similar computation cost compared to references [10,11,12]. Since the handover authentication cost disparity is too big to show in one Figure, no comparison results are shown, as in Figure 12.

### 6.3. Communication Overhead

Communication overhead is also a performance for the authentication scheme since SAGIN has a complex network structure and diverse communication protocol that the communication link is weak and narrow. The authentication schemes are compared based on the same communication parameters in reference [8]. The SSL PUF parameters and others are listed in Table 5. The challenge, c, response, r, error correcting code, u, and Helper data are 511 bits because the SSL PUF chip has a 5% deviation for the same challenge. BCH and Privacy Amplification modules are used to correct the deviation, and full entropy is ensured by the min-entropy of SSL [52].

The communication overhead of the terminal authentication protocol is in Table 6. Similar to the computational overhead in Table 3, the scheme based on SSL needs less communication bandwidth than the schema based on Elliptic Curve Cryptography [7,8,9,10], since the public key transmitted needs more bits. The schema based on a pre-distributed symmetric key [12] uses the minimum overhead. The communication overhead of the handover authentication protocol in Table 7 shows the same conclusion. The schemes in references [8,14] pre-negotiate the handover information while our scheme performs handover dynamically and needs only a few bits compared to the pre-distributed scheme. The results show that our scheme is much more appropriate for handover authentication.

Compared with the communication overhead, the interactive times are also a heavy burden in a protocol. The interactive times of the terminal authentication and handover authentication are listed separately in Table 6 and Table 7. The data shows that our scheme needs fewer times in the terminal authentication procedure. Compared with the handover authentication of [18] and [14] without a server, our scheme behaves normally, because our scheme needs the ground station to switch to a new satellite.

In Section 4.1, GS needs to save N groups (Hash(TID||c),r) for each terminal. In this case, Hash(TID||c) is 128 bits and r is 511 bits. For each terminal, if it needs to authenticate 5000 times in one day, GS needs almost 5000 × 365 × 10 × 80 ≈ 1393 Mb for 10 years of service life. One GS services 1000~2000 terminals easily.

## 7. Conclusions

Many solutions are proposed regarding the high mobility and low latency in SAGIN. Among them, the flexible access requirement is fulfilled over public key cryptography; however, it is time-consuming. Protocols based on pre-shared symmetric keys show excellent performance, but how to share the symmetric keys is a difficult assumption, especially for the enormous and flexible terminals. The inherent security of SSL PUF makes it suitable to be the physical security root for SAGIN. A mutual access authentication and key distribution scheme are proposed based on SSL PUF. The security analysis shows that the protocol achieves unconditionally secure key distribution and can defend against masquerade attacks, replay attacks, and man-in-the-middle attacks. The performance evaluation results show that the proposed protocols have an obvious advantage over the elliptic curve or bilinear pairings-based protocols and settle down the pre-share symmetric key problem in SAGIN in case of little performance cost. Our scheme reveals excellent authentication function and sufficient efficiency. In the future, the group key distribution among SSL PUF chips will be the main focus of our research.

## Figures and Tables

**Table 1:** The Notations and Descriptions.

Notation	Description
T	Terminal
$T_{ssl}$	Terminal with normal SSL PUF
GS	Ground Server
$GS_{CRPs}$	Ground Server with CRPs
$GS_{ssl\_i^{\prime }}$	$\mathrm{Ground}\;\mathrm{Server}\;\mathrm{with}\;\mathrm{matched}\;\mathrm{SSL}\;\mathrm{pair}\;ssl\_i^{\prime }$
Sat	Satellite
$Sat_{ssl\_i}$	The satellite with matched SSL pair ssl_i
N	Number of CRPs of each terminal
$c_{start}$	The starting point of the challenge
c	$\mathrm{Challenge}\;\mathrm{to}\;T_{ssl}$
r	$\mathrm{Response}\;\mathrm{of}\;T_{ssl}\;\mathrm{to}\;c$
u	Error correcting code of r
TID	ID of terminal
$SID_{i}$	ID of satellite
GSID	ID of ground server
$K_{T}$	$\mathrm{The}\;\mathrm{key}\;\mathrm{between}\;T_{ssl}$ $\;\mathrm{and}\;GS_{CRPs}$
$K_{Sat_{i}}$	$\mathrm{The}\;\mathrm{key}\;\mathrm{between}\;Sat_{ssl\_i}$ $\;\mathrm{and}\;GS_{ssl\_i^{\prime }}$
$K_{TSi}$	$\mathrm{The}\;\mathrm{session}\;\mathrm{key}\;\mathrm{between}\;T_{ssl}$ $\;\mathrm{and}\;Sat_{ssl\_i}$
Seed	$\mathrm{The}\;\mathrm{seed}\;\mathrm{of}\;Sat_{ssl\_i}$
Time	Time in the system
Helper data	Helper data for key distribution
SCMD	Pre-switch command
CCMD	Pre-switch confirm command
$Enc_{key}\left(data\right)$	Encrypt data using key
*Hash* (*data*)	Hash function for *data*
*Hmac_key_* (*data*)	Hmac function for data using key
||	Concatenation operation

**Table 2:** Computational cost of typical cryptography operation.

Notation	Description	Computation Time (μs)	
		Terminal/Satellite	Ground Server
$T_{h}$	Hash	2.27	1.25
$T_{puf}$	SSL PUF	0.4	0.4
$T_{BCHE}$	BCH Encoder/Key Gen module	38.85	21.31
$T_{BCHD}$	BCH Decoder/Key Recover module	108.3	52.32
$T_{PA}$	Privacy Amplification	30.12	12.34
$T_{Enc}$	Symmetric Encrypt/Decrypt	2.31	1.12
$T_{KDF}$	Key Distribution Function	2.43	1.25
$T_{prng}$	PRNG or RNG	2.13	1.15
$T_{Xor}$	Bitwise XOR	0.62	0.29
$T_{eccM}$	Point multiplication over an elliptic curve	1020	560
$T_{sign}$	Signature	1202	720
$T_{vs}$	Verify Signature	814	426

**Table 3:** Computational cost of terminal authentication.

Scheme	Terminal (μs)	Satellite (μs)	Ground Server (μs)	Total (μs)
Qi2019	$2T_{eccM}$$\;+\;6T_{h}$ ≈ 2053.62	0	$T_{eccM}$$\;+\;4T_{h}$ ≈ 565	≈2618.62
Cao2021	$2T_{eccM}$$\;+\;5T_{h}$$\;+\;T_{Enc}$$\;+\;4T_{KDF}$ ≈ 2063.38	$T_{KDF}$ ≈ 2.43	$3T_{eccM}$$\;+\;5T_{h}$$\;+\;T_{Enc}$$\;+\;4T_{KDF}$ ≈ 3083.38	≈5149.19
Xu2020	$2T_{eccM}$$\;+\;7T_{h}$ ≈ 2055.89	0	$3T_{eccM}$$\;+\;4T_{h}$ ≈ 1685	≈3740.89
Ostad-Sharif2019	$3T_{eccM}$$\;+\;6T_{h}$ ≈ 3073.62	0	$3T_{eccM}$$\;+\;5T_{h}$ ≈ 1686.25	≈4759.87
Shi2021	$T_{prng}$$\;+\;7T_{h}$$\;+\;3T_{Xor}$ ≈ 19.88	0	$T_{prng}$$\;+\;11T_{h}$$\;+\;5T_{Xor}$ ≈ 16.35	≈36.23
Ours	$3T_{h}$$\;+\;T_{puf}$$\;+\;T_{BCHE}$$\;+\;T_{PA}$$\;+\;T_{Enc}$ ≈ 78.49	$4T_{h}$$\;+\;T_{Enc}$$\;+\;T_{prng}$ ≈ 13.52	$T_{BCHD}$$\;+\;T_{PA}$$\;+\;T_{prng}$$\;+\;3T_{h}$$\;+\;2T_{Enc}$ ≈ 71.8	≈163.81

**Table 4:** Computational cost of handover authentication.

Scheme	Terminal (μs)	Satellite (μs)	Ground Server (μs)	Total (μs)
Xue2020	$3T_{h}$ ≈ 6.81	$3T_{h}$$\;+\;T_{Enc}$ ≈ 9.12	0	≈15.93
Shi2021	$2T_{h}$ ≈ 4.54	$2T_{h}$$\;+\;2T_{PRNG}$ ≈ 6.84	0	≈11.38
Zhu2019	$2T_{h}$ ≈ 4.54	$T_{h}$$\;+\;T_{Enc}$ ≈ 4.58	$T_{h}$ ≈ 2.27	≈11.39
Xue2019	$2T_{eccM}$$\;+\;T_{sign}$ ≈ 3242	$2T_{eccM}$$\;+\;2T_{h}$$\;+\;T_{vs}$ ≈ 3246.54	0	≈6488.54
Cao2021	$T_{eccM}$$\;+\;T_{h}$ ≈ 1022.27	$T_{h}$ ≈ 2.27	0	≈1024.54
Ours	$3T_{h}$$\;+\;T_{Enc}$ ≈ 9.12	$3T_{h}$$\;+\;T_{Enc}$ ≈ 9.12	$2T_{h}$$\;+\;T_{PRNG}$ ≈ 3.65	≈21.89

**Table 5:** Length of protocol parameters.

Parameter	Length (bit)
Symmetric key	128
Public key	3072
Private key	256
Hash/Hmac value	128
Real/Anonymous identify information	128
Random number	128
ID or token for attendance	16
Time stamp, Sequence number	48
Challenge c	511
Response r	511
Error correcting code u	511
Helper data	511
SCMD /CCMD	16

**Table 6:** Communication overhead of terminal authentication.

Scheme	Terminal (bit)	Satellite (bit)	Ground Server (bit)	Total (bit)
Qi2019	1072	560	688	3520
Cao2021	512	1040	1552	3104
Xu2020	1200	560	688	3776
Ostad-Sharif2019	816	1456	768	3040
Shi2021	464	912	448	1824
Ours	703	1311	592	2606

**Table 7:** Communication overhead of handover authentication.

Scheme	Terminal (bit)	Satellite (bit)	Ground Server (bit)	Total (bit)
Xue2020	841	458	0	1299
Shi2021	160	672	160	992
Zhu2019	128	1024	0	1152
Xue2019	1504	128	256 × Number of Terminals	1632 + 256 × Number of Terminals
Cao2021	384	640	3728	4752
Ours	224	1408	832	2464

## Data Availability

Not applicable.
